# Analysis of risk factors for attention deficit hyperactivity disorder among children in Hubei region based on liquid chromatography-tandem mass spectrometry

**DOI:** 10.3389/fpsyt.2026.1922397

**Published:** 2026-08-26

**Authors:** Yingchan Hao, Ruoxi Ran, Ziwei Liu, Dan Lu

**Affiliations:** Department of Laboratory Medicine, Maternal and Child Health Hospital of Hubei Province, Tongji Medical College, Huazhong University of Science and Technology, Wuhan, China

**Keywords:** attention deficit hyperactivity disorder, liquid chromatography-tandem mass spectrometry, nutritional elements, vitamin A, vitamin D

## Abstract

**Objective:**

To quantitatively assess nutritional elements using LC-MS/MS, this study examines the correlations between nutritional elements and vitamins and ADHD in children residing in Hubei Province. Additionally, it explores the associations among various monitoring indicators, thereby providing a reference for the clinical diagnosis and treatment of ADHD.

**Methods:**

The study cohort comprised 354 children diagnosed with ADHD and 350 healthy controls, all of whom attended the Child Health Care Department of Hubei Provincial Maternal and Child Health Hospital between January 2023 and December 2025. Nutritional elements were quantified using ICP-MS, while serum levels of vitamins A and D were measured via LC-MS/MS. Comparative analyses were conducted to identify differences between the groups. Univariate and multivariate logistic regression analyses were employed to evaluate the correlations between these nutritional indicators and ADHD, as well as to identify independent associated factors.

**Results:**

The serum concentrations of vitamins A and D were found to be notably lower in the ADHD group compared to the control group (*P* < 0.05). A logistic regression analysis identified vitamin A and vitamin D were independently inversely associated with ADHD, with statistical significance (*P* < 0.05). An age-stratified subgroup analysis revealed that within the ADHD cohort, children aged 6–8 years exhibited significantly lower levels of both vitamin A and vitamin D compared to the control group. Children aged 9–11 years demonstrated reduced levels of vitamin A, vitamin D, and manganese (Mn), but elevated copper (Cu) levels relative to controls. Furthermore, children aged 12–16 years had significantly lower vitamin D levels than their control counterparts (*P* < 0.05). Among the ADHD subgroups, significant differences were observed in the concentrations of iron (Fe), magnesium (Mg), calcium (Ca), Cu, zinc (Zn), vitamin A, and vitamin D (*P* < 0.05). Spearman correlation analysis revealed a strong association between vitamin A and vitamin D levels in children with ADHD, alongside a significant positive correlation between vitamin A and both Fe and Zn (*P* < 0.05).

**Conclusion:**

Vitamin A and vitamin D deficiencies are prevalent among children with ADHD in Hubei Province, and low levels of these vitamins are independently associated with an increased likelihood of ADHD.

## 1 Introduction

Attention deficit hyperactivity disorder (ADHD) is a prevalent neurodevelopmental disorder primarily characterized by symptoms of inattention, hyperactivity, and impulsive behavior. It typically manifests during childhood and adolescence and frequently continues into adulthood (1), posing significant risks to the physical and mental health of affected individuals. Research indicates that the prevalence of ADHD among children and adolescents is approximately 7.2% (2), with a trend of increasing incidence over time. The etiology and pathogenesis of ADHD remain elusive and are considered highly intricate, potentially involving genetic predispositions, neuroendocrine dysregulation, neurophysiological dysfunction, and other multifaceted factors (3). As research into the etiology of ADHD advances, nutritional interventions (4) have garnered considerable attention as potential adjunctive treatments.

Vitamin D, a steroid hormone synthesized by the human body, exerts its effects by binding to the vitamin D receptor within the nuclear receptor family. It plays a crucial role in metabolism, immune function, inflammatory regulation, and neurodevelopment. Serum 25-hydroxyvitamin D is widely regarded as the most reliable biomarker for evaluating vitamin D status, as it reflects both dietary intake and the metabolic state of vitamin D in the body (5). Research has demonstrated (6) that alterations in the concentration of 25(OH)D within brain cells can influence cytokine regulation and impact various cellular processes, including cell differentiation, intracellular calcium signaling, neurotransmitter release, antioxidant activity, and the expression of genes critical for neuronal function. Vitamin A is a vital micronutrient necessary for numerous physiological processes, including vision regulation, infection resistance, maintenance of epithelial cell integrity, reproductive function, tissue growth, metabolism, and immune response (7). Nutritional elements -including minerals, trace elements, and vitamins are essential for normal physiological functions, enzymatic reactions, and neural signaling in children (8).

In this study, we focused on vitamins A and D because both have been implicated in ADHD pathophysiology, our laboratory has established validated LC-MS/MS protocols for their quantification, and they are routinely screened in our clinical setting. Using LC-MS/MS and ICP-MS, we quantitatively assessed serum levels of vitamins A, D, and calcium, magnesium and eight nutritional elements in children with ADHD and healthy controls from Hubei Province. We aimed to elucidate the associations between these micronutrients and ADHD, explore age-specific patterns, and provide preliminary evidence to inform future nutritional intervention strategies.

## 2 Subjects and methods

### 2.1 Study subjects

A cohort of 354 children diagnosed with ADHD was recruited from the Child Health Care Department at Hubei Provincial Maternal and Child Health Hospital between January 1, 2023, and December 31, 2025, to constitute the ADHD group. Concurrently, a control group comprising 350 healthy children undergoing routine physical examinations in the same department was established. Venous blood samples were collected from all participants on the day of assessment, ensuring no temporal gap between the administration of diagnostic scales and biomarker measurement. Blood samples were collected throughout the study period across all seasons. The inclusion criteria for the ADHD group were as follows: (1) fulfillment of the ADHD diagnostic criteria as outlined in the Diagnostic and Statistical Manual of Mental Disorders, Fifth Edition (DSM-5); (2) age range of 6 to 16 years; (3) absence of prior ADHD treatment history; and (4) normal communication abilities with adequate cognitive and reading skills. All ADHD diagnoses were confirmed by experienced developmental pediatricians in our hospital strictly in accordance with DSM-5 diagnostic criteria. Meanwhile, the SNAP-IV and Conners Parent Rating Scale were used as standardized assessment instruments. Exclusion criteria included: (1) presence of other neuropsychiatric disorders; (2) existence of severe underlying medical conditions, such as significant respiratory, cardiovascular, hematological, or circulatory system diseases; and (3) incomplete clinical data. This study received approval from the Medical Ethics Committee of Hubei Provincial Maternal and Child Health Hospital (Approval No. 2026-013-02).

### 2.2 Methods

#### 2.2.1 Quantification of serum vitamin A and vitamin D via LC-MS/MS

The quantification of serum VA and VD via liquid chromatography-tandem mass spectrometry (LC–MS/MS) followed the identical pretreatment, chromatographic separation and mass spectrometry parameters described in our previously published study focusing on tic disorders in Hubei children (9). Full instrumental setup, internal standard calibration and quality control protocols are detailed in the published manuscript. The laboratory reference ranges used in our institution are as follows: VA (retinol): 300–700 ng/mL; VD (25-hydroxyvitamin D): 20–100 ng/mL (sufficient), <20 ng/mL (deficient).

#### 2.2.2 Quantification of nutritional elements via inductively coupled plasma mass spectrometry

Whole blood nutritional elements quantification by inductively coupled plasma mass spectrometry (ICP-MS) adopted the standardized digestion, dilution and internal standard correction procedures reported in our published tic disorder research (9). We omit repetitive procedural descriptions here to streamline manuscript text.

### 2.3 Statistical analyses

Statistical analyses were performed using SPSS 22.0 and GraphPad Prism 10.0. Normally distributed continuous data are presented as mean ± SD and compared between groups using the t-test. Non-normally distributed data are presented as median (IQR) and compared using the Mann-Whitney U test. Categorical data are expressed as frequencies (percentages) and analyzed using the chi-square test. Vitamins A and D were entered as continuous variables in multivariate logistic regression, adjusting for covariates with *P* < 0.05 in univariate analysis. Model assumptions were checked: *VIF* < 2 confirmed no multicollinearity, and the Box-Tidwell test confirmed linearity of the logit. Spearman’s rank correlation was used to assess associations among vitamins and nutritional elements, with correlation intensity classified as weak (|*r*| < 0.3), moderate (0.3 ≤ |*r*| < 0.5), or strong (|*r*| ≥ 0.5). To control for false positives due to multiple comparisons, the Benjamini-Hochberg FDR correction was applied to the 12 nutritional indicators. Conclusions were based on FDR-adjusted P-values, while raw P-values were shown in figures. Correlation analyses were exploratory and not corrected. A *P*-value of less than 0.05 was considered indicative of statistical significance.

## 3 Results

### 3.1 Comparison of general data

The ADHD cohort comprised 354 children, of whom 296 were male (83.6%) and 58 were female (16.4%). The control cohort included 350 healthy children, with 287 males (82.0%) and 63 females (18.0%). Comparative analyses of gender, place of residence, and age between the two cohorts were conducted using the chi-square test and Mann-Whitney U test, revealing no statistically significant differences (*P* > 0.05) (refer to **Table 1**).

**Table 1:** Comparison of general characteristics between the two groups.

Variables	ADHD (n=354)	Control (n=350)	*χ2/Z*	*P*
Sex (%)			0.323	0.570
Male	296 (83.6)	287 (82.0)		
Female	58 (16.4)	63 (18.0)		
Age	8.13 (7.00,9.42)	8.21 (7.00,9.75)	-0.653	0.514
Residence (%)				
Urban	322 (91.0)	316 (90.3)	0.094	0.759
Rural	32 (9.0)	34 (9.7)		

### 3.2 Correlation analysis of nutritional elements and vitamins in the ADHD group

**Figure 1:**
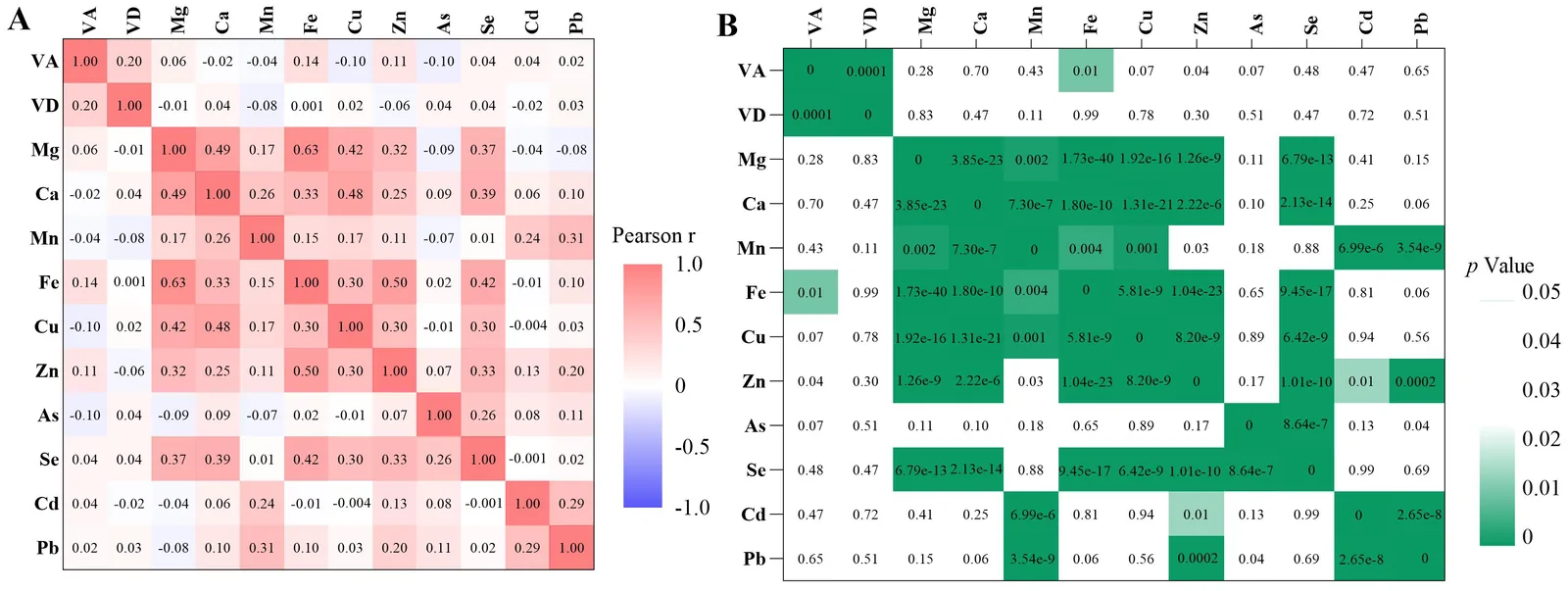
Correlation heatmap of nutritional elements and vitamins in children with ADHD. [r=1 indicates a complete positive correlation; r=−1 indicates a complete negative correlation; Correlation strength was defined as follows: |r| ≥ 0.7, strong; 0.3 ≤ |r| < 0.7, moderate; |r| < 0.3, weak; **(A)** represents the correlation coefficient *r* value; **(B)** represents the statistical *P*-value].

Spearman correlation analysis revealed that vitamin A and vitamin D were positively correlated with each other, with a weak correlation (r = 0.20, P < 0.001). Vitamin A showed weak positive correlations with Fe (r = 0.14, P < 0.05) and Zn (r = 0.11, P < 0.05). Among nutritional elements, Ca exhibited moderate positive correlations with Mg, Fe and Cu (r = 0.33–0.49, all P < 0.001). Mn showed moderate positive correlations with Pb (r = 0.31, P < 0.001). Fe was moderately correlated with Mg, Zn, and Cu (r = 0.30–0.63, all P < 0.001). Cu showed moderate correlations with Mg and Zn (r = 0.30–0.42, all P < 0.001). Se exhibited moderate positive correlations with Mg, Fe, Cu and Zn (r = 0.30–0.42, all P < 0.001).These associations are depicted in **Figure 1**.

### 3.3 Comparison of nutritional elements and vitamin levels between the ADHD and control groups

**Figure 2:**
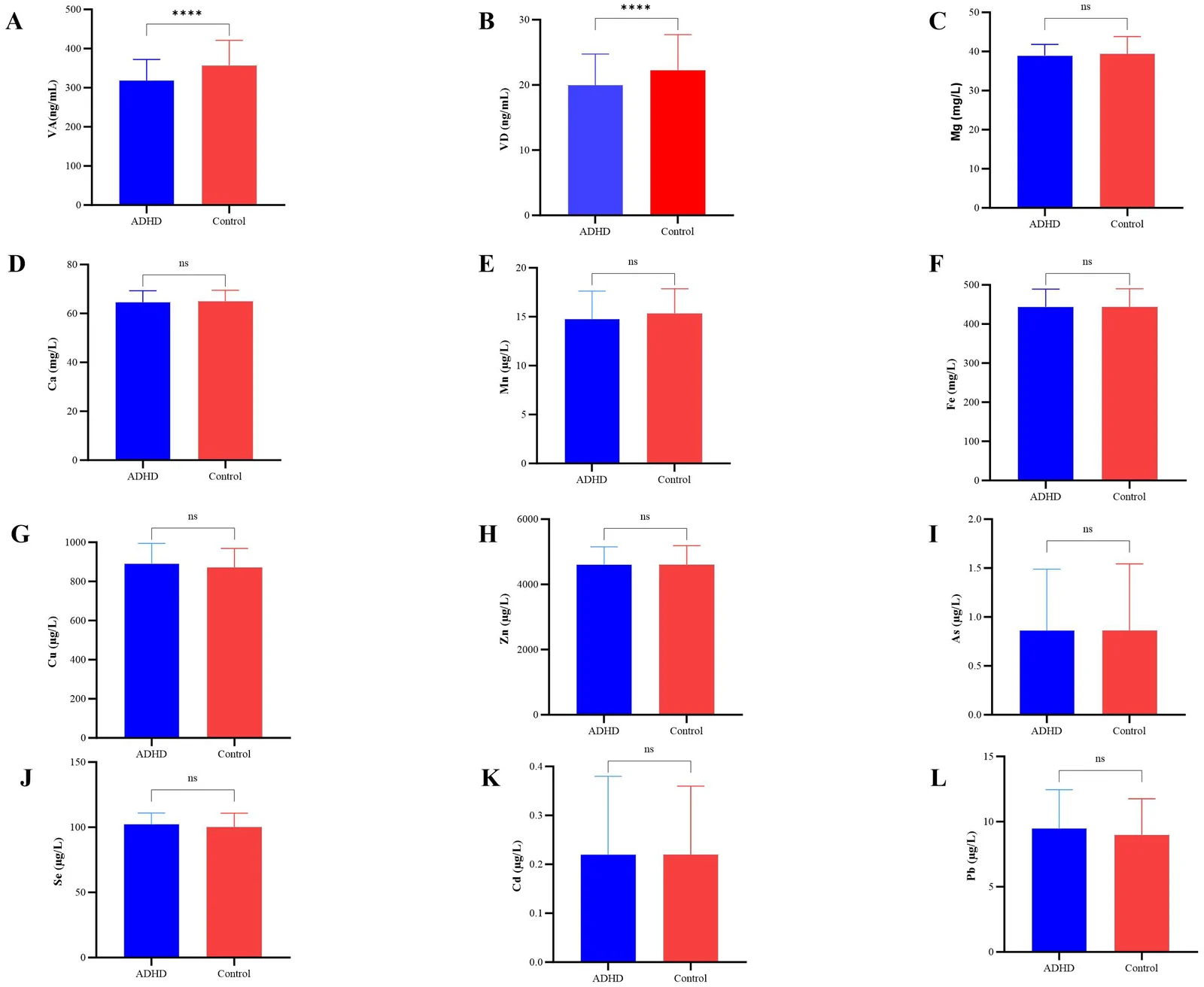
Comparison of nutritional elements and vitamin levels between the two groups (**(A)** VA; **(B)** VD; **(C)** Mg; **(D)** Ca; **(E)** Mn; **(F)** Fe; **(G)** Cu; **(H)** Zn; **(I)** As; **(J)** Se; **(K)** Cd; **(L)** Pb; ****P<0.0001; ns, not significant.).

The uncorrected Mann–Whitney U test revealed that serum Mn and Cu had marginal intergroup differences (*P* = 0.096, *P* = 0.093), while VA and VD were significantly lower in the ADHD group (both *P* < 0.001), as shown in **Figure 2**; After Benjamini–Hochberg FDR correction for the detected indicators, only VA (FDR-adjusted *P* < 0.006) and VD (FDR-adjusted *P* < 0.012) maintained statistical significance. No significant intergroup differences were observed in all tested nutritional elements following FDR correction.

### 3.4 Independent associations between serum vitamins and ADHD status

To further assess whether serum vitamins showed independent associations with ADHD status, both univariate and multivariate logistic regression analyses were conducted. The univariate analysis revealed that for each 1-unit increase in vitamin A and vitamin D levels, the likelihood of ADHD was reduced by 0.5% and 4.6%, respectively (*P* < 0.001). In models 1 and 2, which adjusted for the confounding variables of age and gender, the inverse associations between vitamin A and vitamin D levels and ADHD persisted (*P* < 0.001). These findings suggest that low levels of vitamin A and vitamin D may be associated with an increased likelihood of ADHD, as illustrated in **Table 2**.

**Table 2:** Independent effects of serum vitamins on the risk of ADHD.

Variable	OR	OR,95%CI	*P*
VA	0.995	0.994, 0.997	<0.001
VD	0.954	0.935, 0.974	<0.001
Model 1	0.995	0.993, 0.997	<0.001
Model 2	0.951	0.931, 0.971	<0.001

Model 1 includes vitamin A, age, and gender; Model 2 includes vitamin D, age, and gender.

### 3.5 Stratified analyses of nutritional indicators and ADHD associations across age subgroups

**Figure 3:**
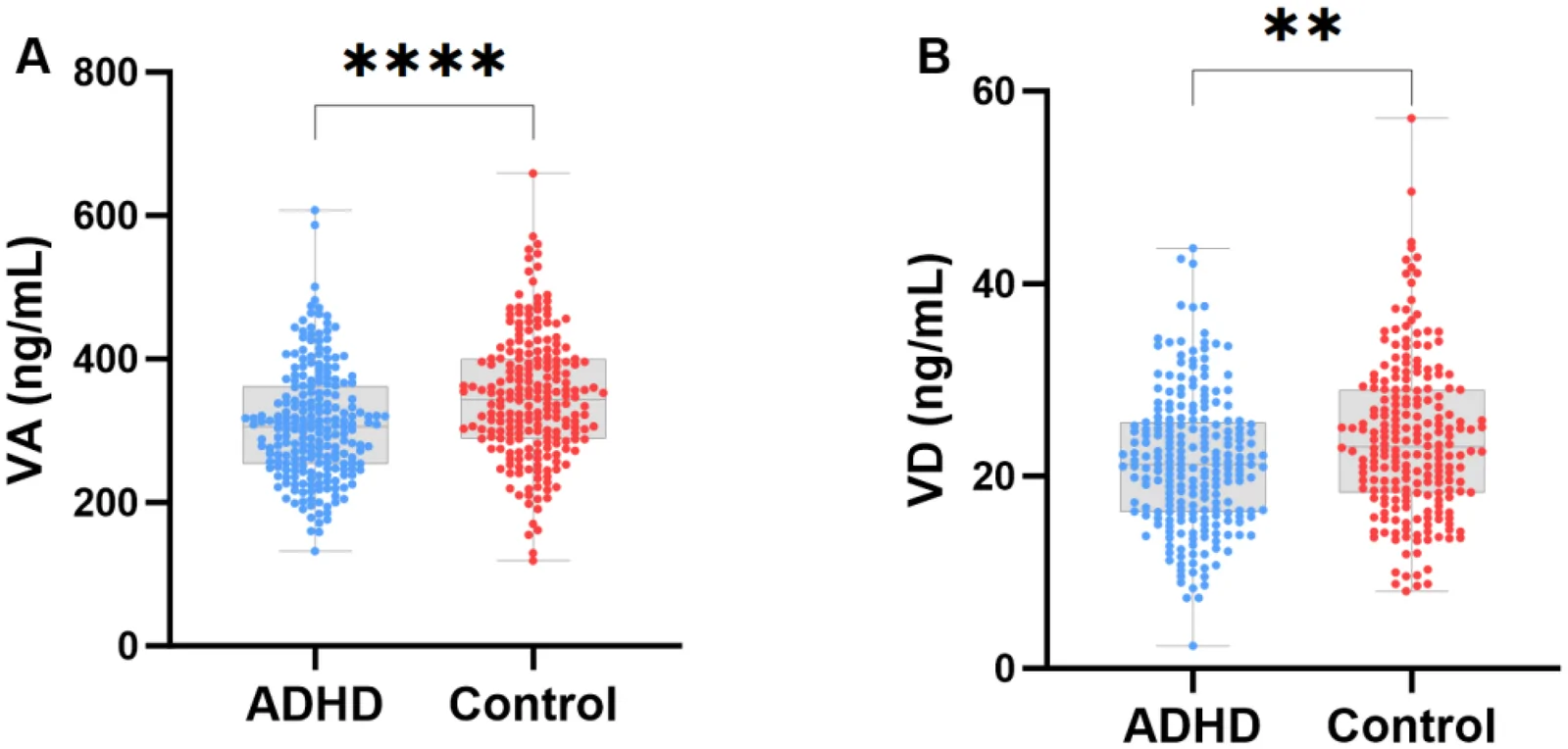
Comparison of VA **(A)** and VD **(B)** levels between two groups within the age subgroup of 6 to 8 years. (****P < 0.0001; **P < 0.01; Full element comparisons are shown in **Supplementary Figure 3**).

**Figure 4:**
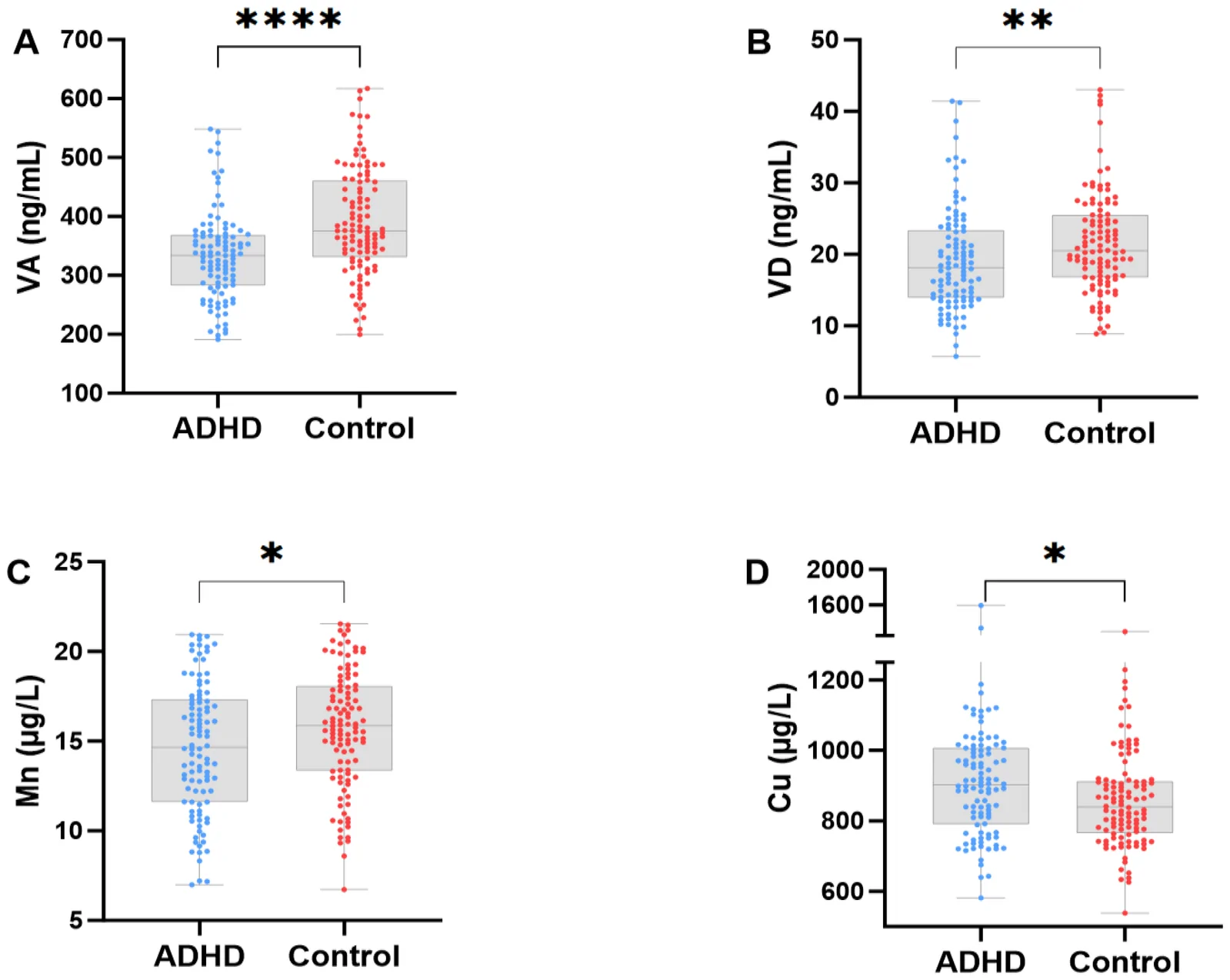
Comparison of VA **(A)**, VD **(B)**, Mn **(C)**, and Cu **(D)** levels between two groups within the age subgroup of 9 to 11 years. (****P < 0.0001; **P < 0.01; *P < 0.05; Full element comparisons are shown in **Supplementary Figure 4**).

**Figure 5:**
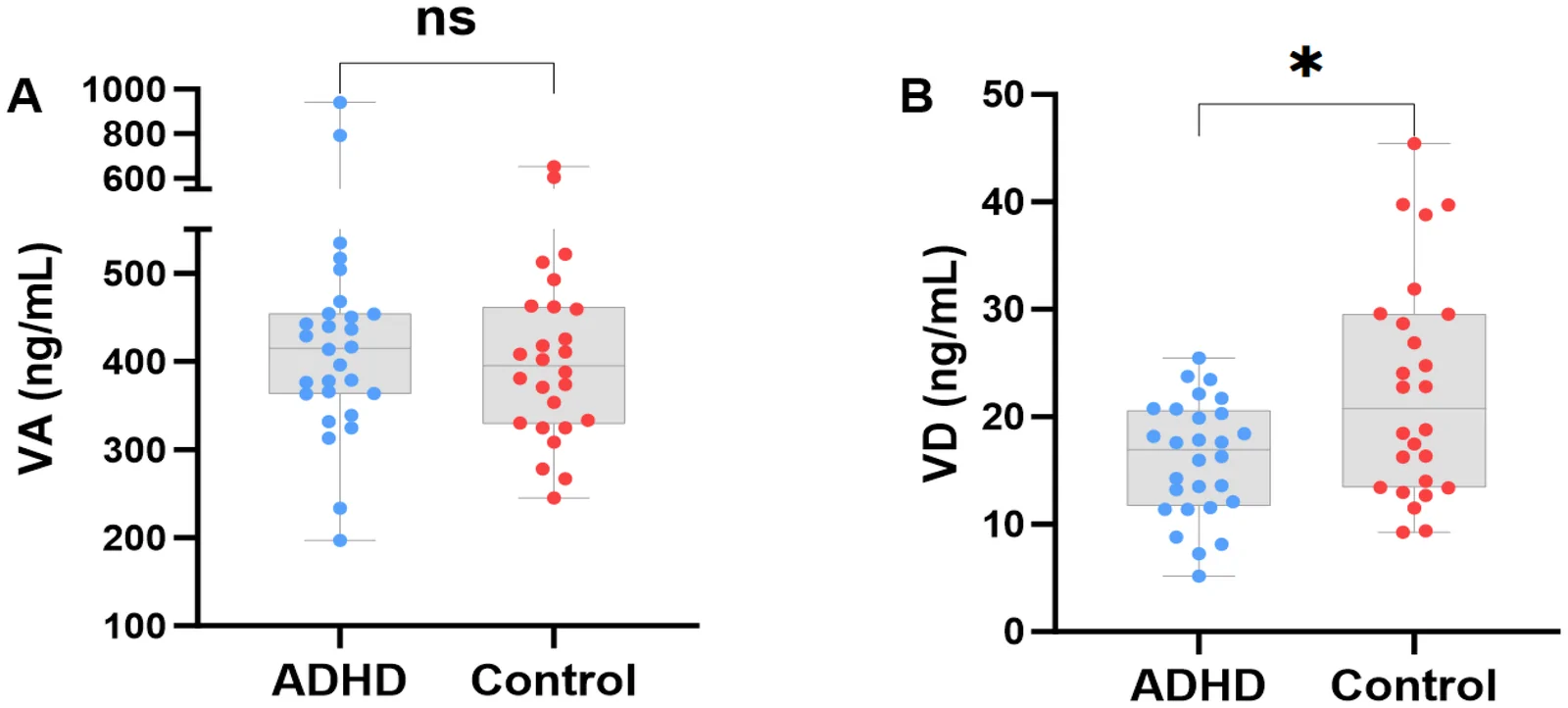
Comparison of VA **(A)** and VD **(B)** levels between two groups within the age subgroup of 12 to 16 years. (*P < 0.05; Full element comparisons are shown in **Supplementary Figure 5**).

The study population was stratified into three distinct age subgroups: 6–8 years, 9–11 years, and 12–16 years based on the time nodes of children’s developmental stages. A statistical evaluation of various nutritional parameters across these groups revealed significant findings. In the ADHD subgroup aged 6–8 years, both vitamin A and vitamin D levels were significantly lower compared to the control group (*P* < 0.05) (refer to **Figure 3**). For the ADHD subgroup aged 9–11 years, there was a marked reduction in the levels of vitamin A, vitamin D, and Mn, alongside an elevated level of Cu, relative to the control group (*P* < 0.05) (refer to **Figure 4**). In the ADHD subgroup aged 12–16 years, a significant decrease in vitamin D levels was observed when compared to the control group (*P* < 0.05) (refer to **Figure 5**). No statistically significant differences were observed in other indicators across the different age subgroups (*P* > 0.05) (refer to **Supplementary Figures 3**-**5**).

### 3.6 Age-stratified comparisons of nutritional indicators among children with ADHD

A one-way ANOVA was conducted to assess the levels of Fe and Mg across three age subgroups within the ADHD cohort, while the Kruskal-Wallis H test, a nonparametric alternative, was employed for the analysis of other nutritional elements, vitamin A, and vitamin D. The findings indicated notable variations in Fe and Mg levels among the different age groups (*P* < 0.05). Furthermore, the Kruskal-Wallis test revealed significant differences in the distributions of Ca, Cu, Zn, vitamin A, and vitamin D levels across the three age subgroups (*P* < 0.05). In contrast, the levels of Mn, As, Se, Cd, and Pb did not exhibit significant differences among the groups (*P* > 0.05). Refer to **Table 3** for detailed results.

**Table 3:** Comparison of nutritional elements and vitamin levels across different age subgroups in the ADHD group.

Variable	6-8 years (n=224)	9-11 years (n=102)	12-16 years (n=28)	χ^2^/*F*/*H*	*P*
Sex(%)					
Male	187(83.5%)	85(83.3%)	24(85.7%)	0.099	0.952
Female	37(16.5%)	17(16.7%)	4(14.3%)		
Fe^1^	437.61 ± 43.13	453.46 ± 47.52	463.93 ± 42.19	7.457	<0.001
Mg^1^	39.00 ± 4.31	40.03 ± 4.32	37.75 ± 4.95	3.630	0.028
Ca^1^	65.33(61.66, 69.77)	64.33(60.96, 69.66)	61.99(57.52, 64.84)	9.630	0.008
Mn^2^	14.87(11.64, 18.03)	14.67(11.63, 17.31)	13.50(10.37, 15.98)	4.093	0.129
Cu^2^	897.43(819.31, 999.78)	902.71(791.80, 1005.60)	815.15(719.84, 879.57)	13.793	0.001
As^2^	0.86(0.45, 1.55)	0.86(0.46, 1.37)	0.86(0.58, 1.92)	0.718	0.698
Zn^2^	4512.30(4067.10, 5034.60)	4749.05(4358.10, 5279.20)	4916.90(4058.00, 5482.75)	10.325	0.006
Se^2^	102.24(91.33, 110.05)	104.31(96.21, 114.94)	99.13(89.45, 109.09)	4.970	0.083
Cd^2^	0.22(0.12, 0.38)	0.22(0.12, 0.38)	0.26(0.20, 0.38)	2.497	0.287
Pb^2^	9.45(7.13, 12.74)	9.69(6.84, 11.47)	8.97(7.42, 10.52)	1.022	0.600
VA^3^	305.86(254.02, 362.60)	333.80(283.94, 367.62)	415.16(363.63, 454.21)	33.725	<0.001
VD^3^	21.21(16.27, 25.57)	18.13(14.01,23.28)	16.95(11.85,20.52)	20.809	<0.001

Unit definitions: 1 = mg/L, 2 =μg/L, 3 = ng/mL.

### 3.7 Sensitivity analysis after adjusting for additional confounders

To evaluate the robustness of our findings against potential unmeasured confounding, we conducted a sensitivity analysis in a subset of participants for whom additional covariate data (gender, age, residence, BMI, outdoor time, sleep disorders, parenting style, family cohesion, and parental highest education level) were successfully retrieved via medical records and telephone follow-up.

As shown in **Supplementary Table 1**, there were no significant differences in sex, age, residence, BMI, outdoor time, sleep disorders, parenting style, family cohesion, or parental highest education level between the two groups in this subset (*P* > 0.05), whereas VA and VD levels remained significantly lower in the ADHD group (*P* = 0.023 and *P* < 0.001, respectively).

Multivariate logistic regression analysis adjusting for all the above covariates revealed that VA (*P* = 0.012) and VD (*P* < 0.001) remained independently associated with ADHD (**Supplementary Table 2**). Notably, the effect sizes and directions were consistent with those observed in the primary analysis, suggesting that our main findings are unlikely to be substantially confounded by these lifestyle and socioeconomic variables.

## 4 Discussion

ADHD is a prevalent neurodevelopmental condition in childhood, characterized by core symptoms of inattention, hyperactivity, and impulsivity. The etiology of ADHD is multifaceted, involving genetic, neurodevelopmental, nutritional, and other diverse factors, though its pathogenesis remains incompletely understood (10). Micronutrients play a crucial role in the development of the nervous system, neurotransmitter synthesis, and the maintenance of neural function homeostasis in children, and are significantly associated with the onset and progression of ADHD. Consequently, they have emerged as a focal point of current research into the auxiliary etiology and intervention strategies for ADHD (11). This study employed LC-MS/MS and ICP-MS technologies to quantitatively assess serum levels of vitamin A, vitamin D, calcium, magnesium and eight trace elements in children diagnosed with ADHD and in healthy controls from Hubei Province. The research systematically analyzed the correlation between each nutritional parameter and the incidence of ADHD, including age-stratified characteristics, with the aim of elucidating the nutritional abnormalities present in children with ADHD in this region. It is important to emphasize that our findings are observational and hypothesis-generating; they do not support clinical.

Our findings indicate that serum concentrations of vitamin A and vitamin D in the ADHD group were significantly lower than those observed in the healthy control group, with statistical significance. Multivariate logistic regression analysis further demonstrated that vitamin A and vitamin D were independently inversely associated with ADHD in children; higher concentrations of these vitamins were associated with a lower likelihood of ADHD. These results are in alignment with existing domestic and international research (12, 13). Vitamin D, a crucial neuroregulatory steroid hormone, influences neuronal differentiation, neurotransmitter release, calcium signaling, and the balance of oxidative stress in the brain by activating the vitamin D receptor. Deficiency has been associated with impaired neurodevelopment in the cerebral cortex and hippocampus, diminish attention and behavioral regulation, and lead to ADHD-related behavioral abnormalities (14). Similarly, vitamin A, through the retinoic acid receptor-mediated signaling pathway, is involved in forebrain development, synaptic plasticity, and the maintenance of cognitive function, while also regulating the homeostasis of trace element metabolism. Concurrent deficiency of both vitamins is associated with prefrontal cortex circuit dysfunction (13). Spearman correlation analysis in this study showed a significant synergistic correlation between vitamin A and vitamin D in children with ADHD, and a positive correlation between vitamin A and Fe and Zn levels, suggesting that vitamins A and D, through synergistic effects and regulation of trace element metabolism, jointly maintain normal nervous system development. Concurrent deficiency of both vitamins is independently associated with an increased likelihood of ADHD among children in Hubei Province. Therefore, when assessing the nutritional status of children with ADHD, we should not look at a single indicator but should comprehensively analyze the nutritional spectrum. Whether combined supplementation offers additional benefits over single-nutrient intervention requires further investigation through well-designed prospective studies or randomized controlled trials.

The age-stratified subgroup analysis revealed that the influence of nutritional factors on childhood ADHD exhibits significant age-specific characteristics, with notable differences in the nutritional disorder profiles among various age groups. In young children with ADHD aged 6–8 years, there was a reduction in serum vitamin A and D levels, without apparent abnormalities in nutritional elements. This finding suggests that the onset of ADHD in this age group was primarily associated with deficiencies in vitamins A and D. Children within this developmental stage are in a critical period of rapid nervous system development, characterized by heightened demand and sensitivity to vitamins A and D. Deficiencies in these vitamins likely disrupt neural development homeostasis, which correlates with the presentation of ADHD symptoms. Conversely, in the middle school-age subgroup (9–11 years), the nutritional abnormalities were more complex. In addition to reduced levels of vitamins A and D, there were also decreased Mn levels and increased Cu levels. This suggests that with advancing age, trace element metabolic imbalances alongside vitamin deficiencies correlate with ADHD presentation and symptom severity. Mn serves as a cofactor for numerous enzymes, such as glutamine synthetase and arginase, playing a crucial role in the synthesis of brain antioxidants and neural signal transduction. A deficiency in Mn can exacerbate oxidative damage to neuronal cells. This study identified reduced serum Mn levels in children with ADHD in Hubei Province, a finding that contradicts previous research (15). The observed discrepancies may be attributed to variations in detection matrices, exposure levels, regional environmental factors, and age groups, indicating that the relationship between manganese and ADHD is not straightforwardly linear but is influenced by multiple factors. Consequently, further large-scale studies are warranted for confirmation. Cu is involved in the regulation of dopamine β-hydroxylase activity, and elevated Cu levels correlate with oxidative stress in brain tissue, impaired neuronal function, and more severe inattention and hyperactivity symptoms (16). The imbalance of Mn and Cu observed at specific ages suggests that trace element disorders associated with ADHD may be age-dependent. Future research should consider conducting longitudinal follow-up studies across different developmental stages. Within the adolescent subgroup (ages 12–16), individuals diagnosed solely with ADHD exhibited significantly reduced levels of vitamin D, whereas levels of vitamin A and nutritional elements did not significantly differ from those observed in the control group. It is hypothesized that adolescents in this age range typically maintain a balanced diet and stable trace element metabolism. However, due to factors such as heightened academic pressure, limited outdoor activity, and insufficient exposure to sunlight, they are susceptible to chronic vitamin D deficiency. This deficiency correlates with persistent ADHD symptoms in adolescents.

A comparative analysis of various age subgroups within the ADHD cohort revealed statistically significant differences in the distribution of Fe, Mg, Ca, Cu, Zn, vitamin A, and vitamin D levels, indicating that nutritional metabolic disorders in children with ADHD exhibit dynamic age-related evolution. As children age, there is an observed increase in vitamin A levels, whereas vitamin D levels tend to decrease. This inverse relationship may be attributed to reduced outdoor activities and insufficient sunlight exposure during adolescence. Whether vitamin D supplementation could be beneficial for adolescents with ADHD warrants investigation in future prospective studies. Additionally, Fe and Zn levels were found to increase with age, potentially due to heightened intake demands associated with accelerated growth during adolescence. However, it remains to be determined whether children with ADHD exhibit differences in dietary intake compared to their healthy peers, possibly due to behavioral issues such as selective eating or irregular eating patterns, necessitating further dietary survey investigations. Furthermore, the analysis of heavy metals, including Pb, Cd, and As, revealed no significant differences among the groups, indicating that environmental exposure to these heavy metals is not a major contributing factor for ADHD in children residing in Hubei Province.

The intergroup analysis demonstrated no significant differences in conventional nutritional elements such as Fe, Mg, Ca, and Zn between the two groups, with abnormalities observed only in specific age subgroups. This suggests that isolated trace element imbalances are not independently associated with ADHD status. Instead, age-specific periodic imbalances and combined nutritional disturbances correlate with the presence and severity of ADHD symptoms. Additionally, there are extensive positive associations between vitamins and nutritional elements, as well as among the nutritional elements themselves, creating a metabolic network of mutual regulation. Disruption of a single indicator can initiate a cascade of metabolic imbalances, thereby disturbing the homeostasis necessary for nervous system development. This phenomenon is a significant factor contributing to the heterogeneity of nutritional abnormality characteristics observed in children with ADHD across different age groups.

Beyond statistical significance, the clinical relevance of the observed vitamin differences merits discussion. According to the Chinese Expert Consensus (2024), the median vitamin A and D levels in our ADHD group fell within the low-normal to marginal deficiency range, suggesting increased risk of nutritional insufficiency rather than overt clinical deficiency. This distinction matters, as marginal deficiencies may subtly affect neurotransmitter regulation, synaptic plasticity, and oxidative stress.

Emerging evidence (17) suggests vitamin D supplementation may improve ADHD symptoms, but optimal protocols remain unclear. Vitamin A supplementation evidence is limited. Given our observational design, we cannot infer causality or recommend routine supplementation. Our findings are hypothesis-generating and warrant prospective studies and intervention trials to determine whether correcting marginal deficiencies yields clinically meaningful symptom improvement.

### 4.1 Limitations and future directions

Several limitations of this study should be acknowledged. First, this is a single-center retrospective study, which may limit the generalizability of our findings. Second, the retrospective design precludes any causal inferences; therefore, our results should be interpreted as associational and hypothesis-generating rather than causal. Third, while we acknowledge that important confounders such as dietary intake, vitamin supplementation, and precise sunlight exposure duration were not available in the primary cohort, we performed a rigorous sensitivity analysis by retrieving available data on BMI, outdoor activity time, sleep disorders, parenting style, family cohesion, and parental education level via medical records and telephone follow-up for a subset of participants. Encouragingly, after adjusting for all these additional covariates, the protective associations of VA and VD with ADHD remained statistically significant and the effect estimates were highly consistent with the primary analysis. This consistency suggests that residual confounding by these variables is unlikely to fully explain our observed associations. Nevertheless, due to the smaller sample size of this sensitivity subset and the inability to retrieve all lifestyle variables (e.g., detailed dietary records, actual sunlight exposure hours) for the entire cohort, we cannot entirely exclude the possibility of residual confounding. Future multicenter, prospective cohort studies with comprehensive nutritional and lifestyle assessments are warranted to validate these findings. Finally, we have systematically rephrased all causal language throughout the manuscript, replacing “risk factors” with “independently associated factors” to reflect the observational nature of our study accurately.

## 5 Conclusion

In summary, deficiencies in vitamins A and D are independently associated with an increased likelihood of ADHD patients in Hubei Province; both vitamins show significant inverse associations with ADHD status. The nutritional metabolic disorders observed in children with ADHD exhibit considerable age specificity: younger children (6–8 years) predominantly experience deficiencies in vitamins A and D; school-aged (9–11 years) children display combined metabolic abnormalities involving nutritional elements such as Mn and Cu; and adolescents (12–16 years) primarily suffer from persistent vitamin D deficiency. In clinical practice, it is imperative to develop individualized nutritional screening and intervention strategies tailored to the specific nutritional abnormalities characteristic of different age groups. This includes targeted supplementation of vitamins A and D, as well as the correction of periodic imbalances in nutritional elements. By implementing nutritional adjunctive interventions, it is possible to enhance the neurodevelopmental status of children and alleviate the clinical symptoms of ADHD, thereby offering a novel clinical reference for the comprehensive prevention and management of ADHD in children within Hubei Province.

## Data Availability

The original contributions presented in the study are included in the article/**Supplementary Material**. Further inquiries can be directed to the corresponding author.
