# Supplementary material for: Analysis of risk factors for attention deficit hyperactivity disorder among children in Hubei region based on liquid chromatography-tandem mass spectrometry
Source: Front Psychiatry. 2026 Aug 26;17:1922397. doi: 10.3389/fpsyt.2026.1922397 (PMC13553830; doi:10.3389/fpsyt.2026.1922397)
Supplement: Supplementary file 1 [file DataSheet1.pdf]

Supplementary Table S1 Baseline characteristics of the sensitivity cohort.

Variables	ADHD (n=87)	Control (n=95)	*χ2/Z*	*P*
Sex (%)				
Male	69 (79.3)	75 (78.9)	0.004	0.952
Female	18 (20.7)	20 (21.1)		
Age	8.42(7.00,10.00)	8.25 (7.17,10.00)	-0.068	0.946
Residence (%)				
Urban	77 (88.5)	88 (92.6)	0.913	0.339
Rural	10(11.5)	7 (7.4)		
VA[1]	325.04(281.87,371.36)	356.84(310.64,408.92)	-2.869	0.004
VD[1]	16.65(14.06,22.62)	22.14(18.37,28.44)	-4.045	<0.001
BMI	17.10(16.57,17.75)	17.45(16.69,18.13)	-1.417	0.157
Outdoor time (%)				
≤120min	77 (88.5)	82(86.3)	0.197	0.657
＞120min	10(11.5)	13(13.7)		
sleep disorders (%)				
Yes	8(9.2)	11(11.6)	0.276	0.599
No	79(90.8)	84(88.4)		
Parenting style (%)				
Harsh punishment	1(1.1)	2(2.1)	0.256	0.613
Rational lecturing	86(98.9)	93(97.9)		
Family cohesion (%)				
poor	1(1.1)	1(1.1)	0.004	0.950
good	86(98.9)	94(98.9)		
Parental highest education(%)				
≤Junior high	4(4.6)	5(5.3)	0.098	0.952
High school	14(16.1)	14(14.7)		
≥Bachelor’s degree	69(79.3)	76(80.0)		

Note: Unit definitions: 1 = ng/mL, BMI, body mass index

Supplementary Table S2 Multivariate logistic regression analysis for ADHD in the sensitivity cohort.

Variable	OR	OR,95%CI	*P*
VA	0.994	0.990, 0.998	0.004
VD	0.911	0.869, 0.955	<0.001
Model 1	0.994	0.990, 0.999	0.012
Model 2	0.908	0.862, 0.955	<0.001

Note: Model 1 = Adjusted model for VA; Model 2 = Adjusted model for VD; Adjusted covariates: gender, age, residence, BMI, outdoor time, sleep disorders, parenting style, family cohesion, parental highest education.

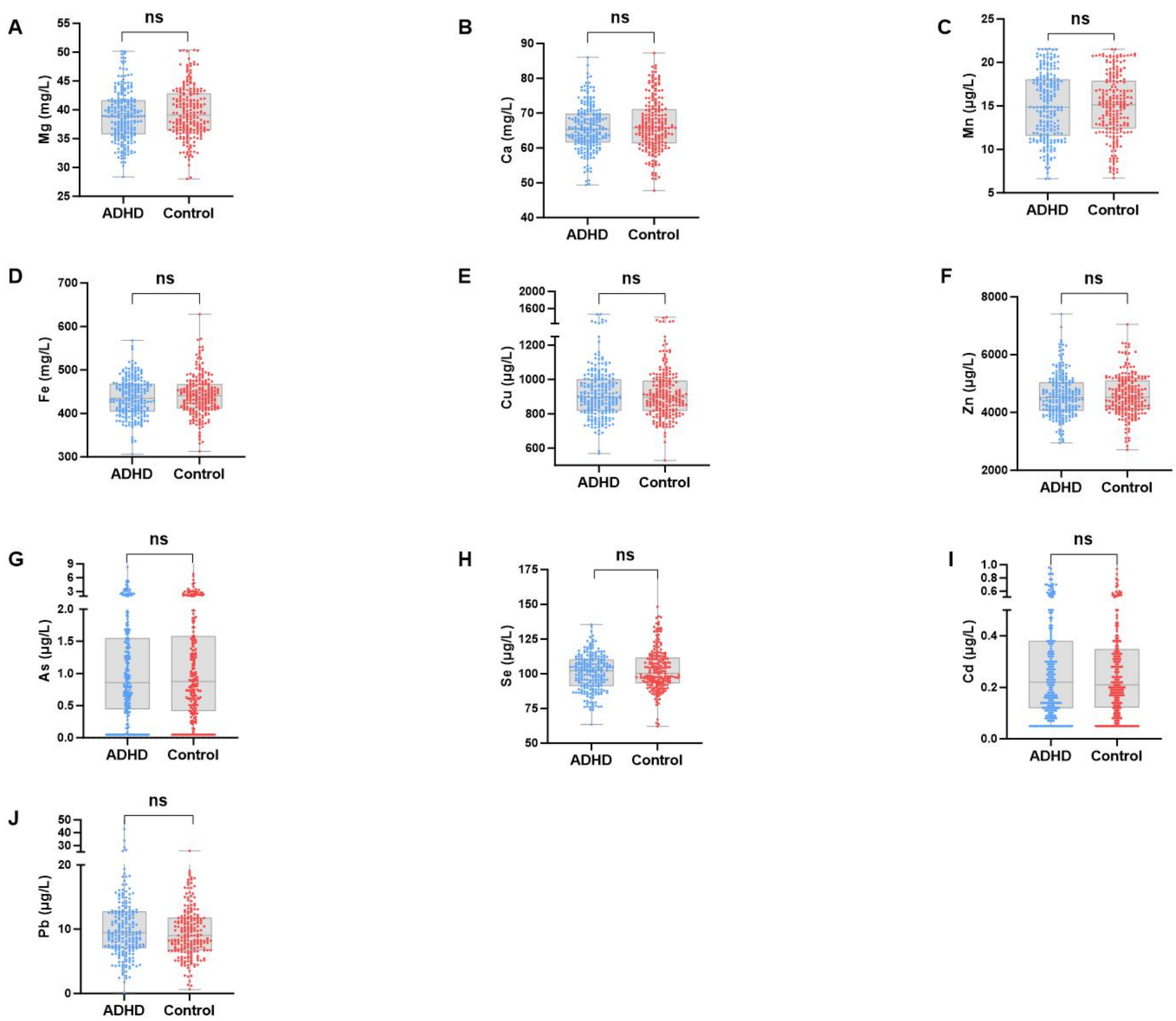

Supplementary Figure S3 Comparison of nutritional elements levels between two groups within the age subgroup of 6 to 8 years.(Note: A: Mg; B: Ca; C: Mn; D: Fe; E: Cu; F: Zn; G: As; H: Se; I: Cd; J: Pb; ns: not significant.)

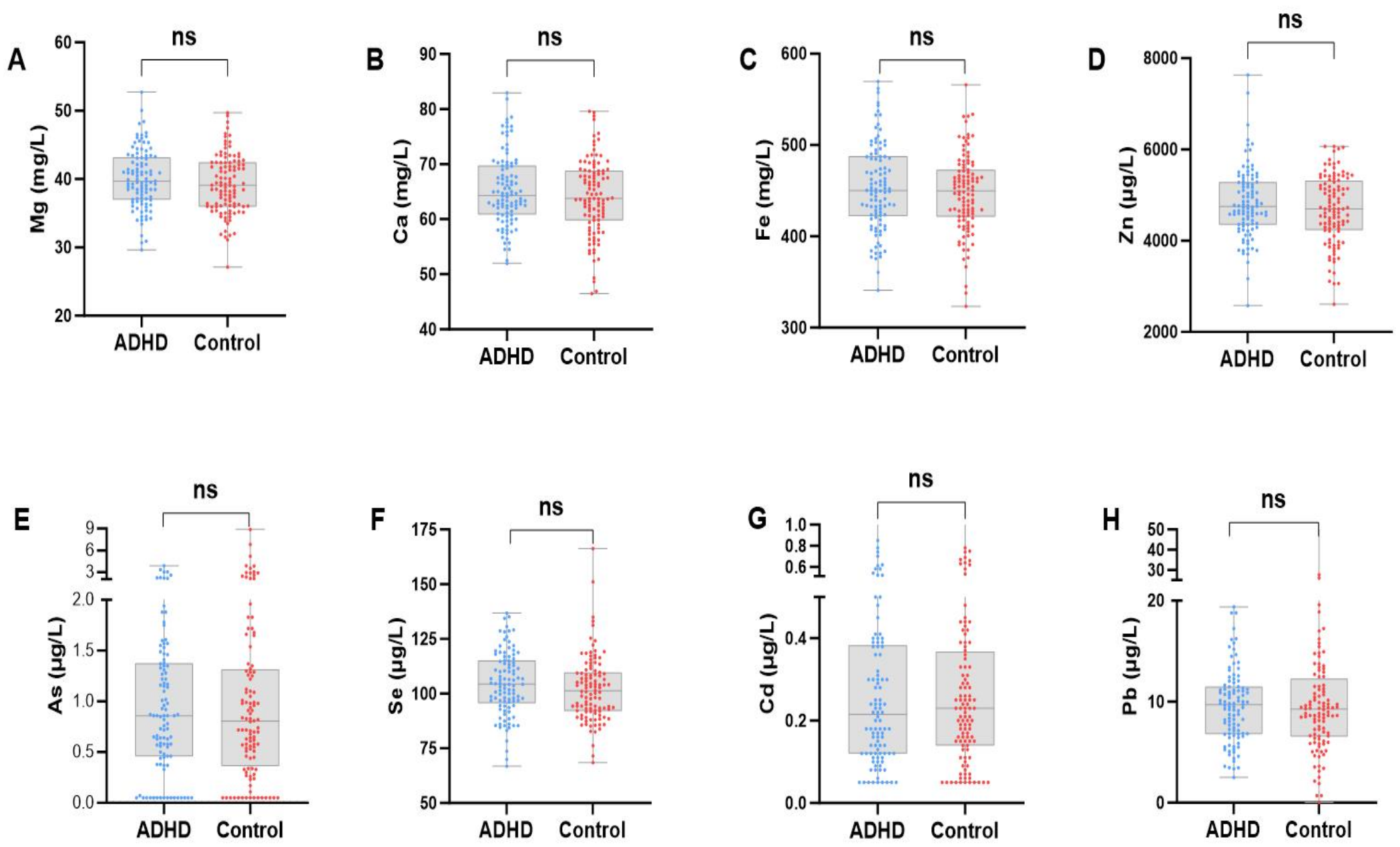

Supplementary Figure S4 Comparison of nutritional elements levels between two groups within the age subgroup of 9 to 11 years. (Note: A: Mg; B: Ca; C: Fe; D: Zn; E: As; F: Se; G: Cd; H: Pb; ns: not significant.)

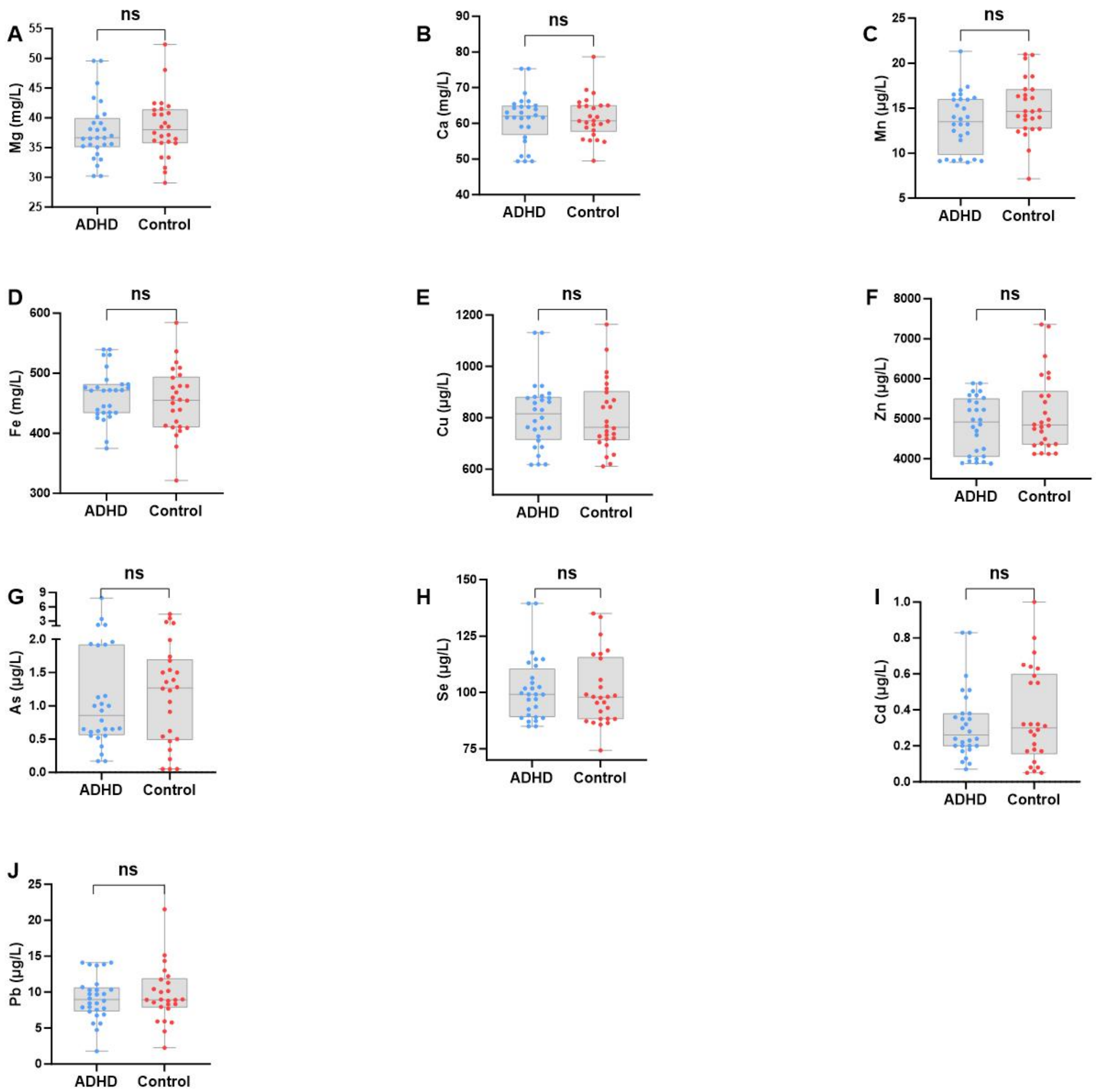

Supplementary Figure S5 Comparison of nutritional elements levels between two groups within the age subgroup of 12 to 16 years. (Note: A: Mg; B: Ca; C: Mn; D: Fe; E: Cu; F: Zn; G: As; H: Se; I: Cd; J: Pb; ns: not significant.)
